# The adhesion G protein-coupled receptor F5 is a crucial regulator of CD4^+^ T cell function: Implications for colon cancers and colitis progression

**DOI:** 10.1016/j.gendis.2026.102107

**Published:** 2026-02-23

**Authors:** Damian Jacenik, Marta Zielińska, Federica Ungaro, Silvio Danese, Ellen J. Beswick, Jakub Fichna

**Affiliations:** a Department of Cytobiochemistry, Faculty of Biology and Environmental Protection, University of Lodz, 141/143 Pomorska St., Lodz 90-236, Poland; b Department of Biochemistry, Faculty of Medicine, Medical University of Lodz, 5 Mazowiecka St., Lodz 92-215, Poland; c Division of Immunology, Transplantation and Infectious Disease, Department of Gastroenterology and Digestive Endoscopy, IRCCS Ospedale San Raffaele Hospital, 60 Via Olgettina St., Milan 20132, Italy; d Division of Molecular Medicine, Department of Internal Medicine, University of New Mexico Health Sciences Center, 1 University of New Mexico St., Albuquerque, NM 87131, United States

Immunosuppression is a well-known phenomenon responsible for the failure of anti-cancer treatment approaches. The reduced ability of the immune response against cancer cells is driven by specialized immune cells such as myeloid-derived suppressive cells, tumor-associated macrophages and neutrophils, or regulatory T cells, which favor the progression of cancers.^1^ Experimental studies have identified numerous factors in effector immune cells that can abolish their activity against cancer cells. Here, we postulated that the adhesion G protein-coupled receptor F5 (ADGRF5) is an important regulator of colon cancer progression by affecting the immune response. To investigate the therapeutic potential of ADGRF5, we employed wild type (WT) and *Adgrf5* knockout (KO) mice and the murine model of colon tumors (Supplementary data). We identified that mice with *Adgrf5* KO developed smaller colon tumors, which were more prone to T cell infiltration, as evaluated by profiling of CD3E marker expression (Fig. 1A and B). By employing an *ex vivo* approach, we indicated that *Adgrf5* KO in CD4^+^ T cells reprograms their functions and intensifies their action against cancer cells. Compared to WT CD4^+^ T cells, *Adgrf5* KO CD4^+^ T cells overexpressed lytic factors such as granzyme B (GZMB) and perforin 1 (PRF1) and pro-inflammatory cytokines such as interferon γ (IFN-γ), and manifested by limited production of anti-inflammatory interleukins (ILs) and enhanced secretion of pro-inflammatory factors such as IL-2 and IFN-γ (Fig. 1C and D). Our analysis of curves representing the growth of allograft tumors derived from MC38 cells revealed a significantly lower volume of colon tumors in mice treated with *Adgrf5* KO CD4^+^ T cells than in control animals and mice treated with WT CD4^+^ T cells (Fig. 1E). The anti-cancer effects of *Adgrf5* KO CD4^+^ T cells were confirmed employing an *in vitro* approach and Hoechst 33342 staining, noting that the expression of ADGRF5 determines the tumor killing function of CD4^+^ T cells (Fig. 1F). In particular, IFN-γ is one of the major cytokines responsible for the induction of tumor cell apoptosis, which limits tumor growth.^2^ As shown in Figure 1G, the administration of anti-IFN-γ antibody abolished the anti-cancer effect of *Adgrf5* KO CD4^+^ T cells, whereas treatment with anti-IFN-γ antibody alone significantly boosted the growth of allograft tumors. According to our findings, ADGRF5 is the major regulator of the phenotype of CD4^+^ T cells, and its depletion switches the immune response mediated by CD4^+^ T cells into pro-inflammatory and anti-cancer stages.

**Figure 1:**
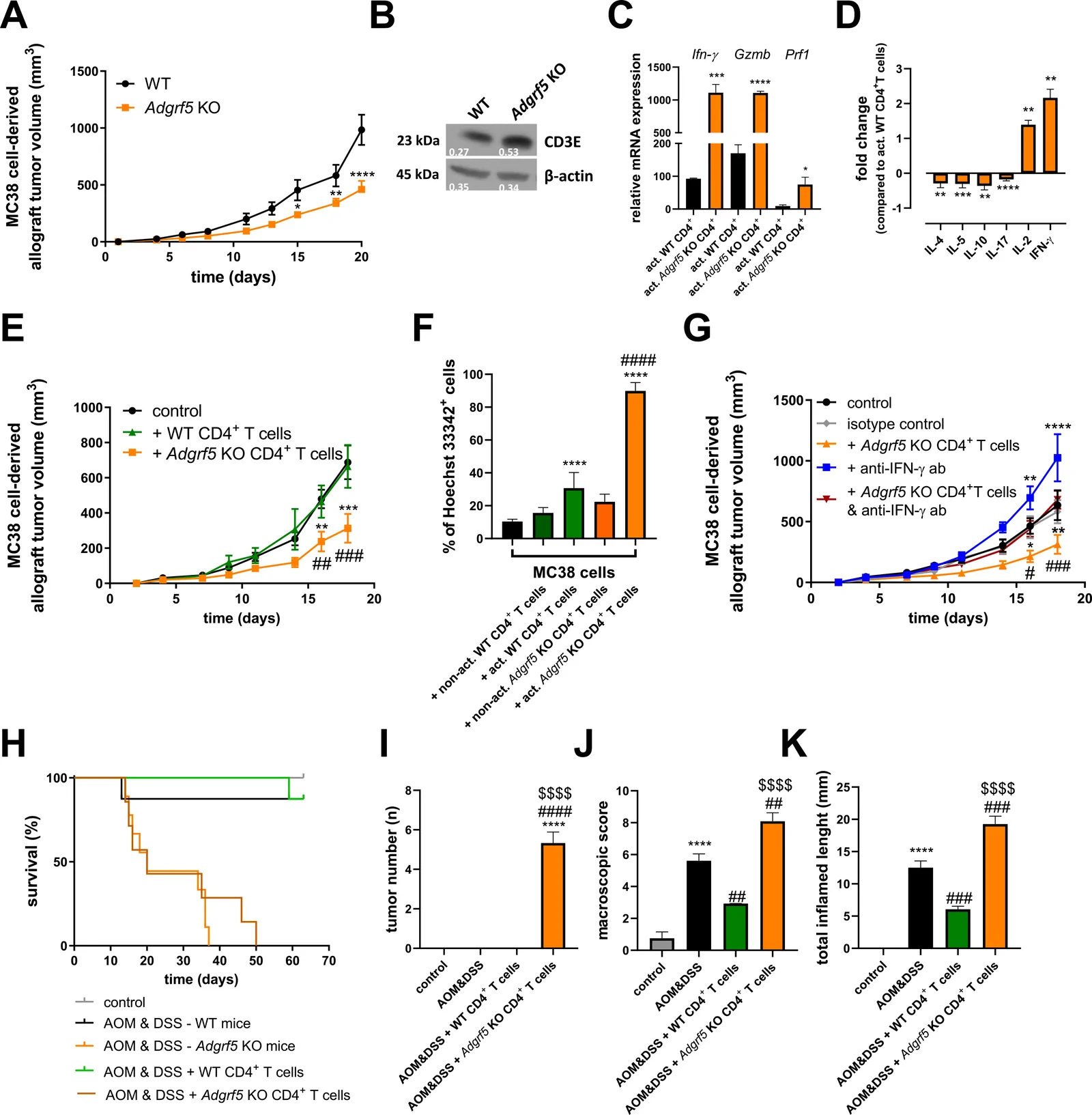

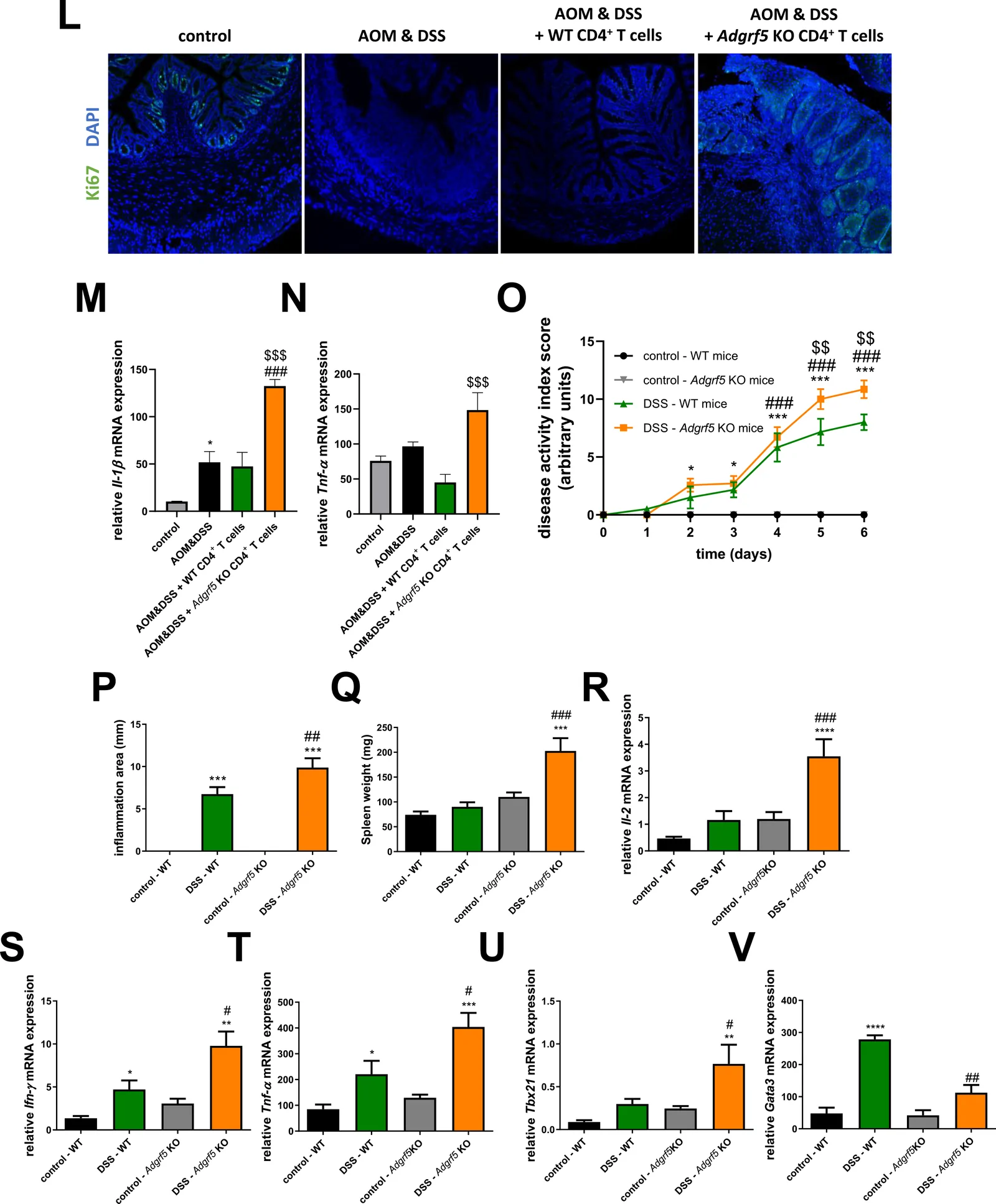
ADGRF5 affects progression of colon cancer and colitis. The growth profile **(A)** of MC38 cells-derived allografts in WT and *Adgrf5* KO mice (*n* = 15 mice per group). Representative images **(B)** showing the protein level of CD3E and β-actin in MC38 cells-derived allografts obtained from WT and *Adgrf5* KO mice. The expression of *Ifn-γ*, *Gzmb* and *Prf1***(C)** at the mRNA level in activated WT and *Adgrf5* KO CD4^+^ T cells (*n* = 10 samples per group). The concentration of IL-2, IL-4, IL-5, IL-10 and IL-17 as well as IFN-γ **(D)** released from activated WT and *Adgrf5* KO CD4^+^ T cells (*n* = 10 samples per group). Data are presented as means ± SEM; ∗*p* < 0.05, ∗∗*p* < 0.01, ∗∗∗*p* < 0.001, ∗∗∗∗*p* < 0.0001. The growth profile **(E)** of MC38 cells-derived allografts in WT mice untreated and treated with WT or *Adgrf5* KO CD4^+^ T cells (*n* = 10 mice per group). The percent of Hoechst 33342^+^ MC38 cells **(F)** obtained from co-culture of MC38 cells with activated WT and *Adgrf5* KO CD4^+^ T cells (*n* = 10 samples per group). The growth profile **(G)** of MC38 cells-derived allografts in WT mice untreated and treated with *Adgrf5* KO CD4^+^ T cells or anti-IFN-γ antibodies or a mixture of both (*n* = 10 mice per group). Data are presented as means ± SEM; ∗*p* < 0.05, ∗∗*p* < 0.01, ∗∗∗*p* < 0.001, ∗∗∗∗*p* < 0.0001 *vs.* control; ^#^*p* < 0.05, ^##^*p* < 0.01, ^###^*p* < 0.001, ^####^*p* < 0.0001 *vs.* WT CD4^+^ T cells or activated CD4^+^ T cells or *Adgrf5* KO CD4^+^ T cells & anti-IFN-γ antibodies. The survival **(H)** of WT untreated mice; WT and *Adgrf5* KO mice with induced colitis-associated colon cancer; WT mice with induced colitis-associated colon cancer treated with WT or *Adgrf5* KO CD4^+^ T cells (*n* = 15 mice per group). The number of tumors **(I)**, macroscopic damage score **(J)** and total inflamed length **(K)** of WT untreated mice, WT mice injected with AOM and treated with DSS and WT mice with induced colitis-associated colon cancer treated with WT or *Adgrf5* KO CD4^+^ T cells (*n* = 10 mice per group). Data are presented as means ± SEM; ∗∗∗∗*p* < 0.0001 *vs.* control; ^##^*p* < 0.01, ^###^*p* < 0.001, ^####^*p* < 0.0001 *vs.* AOM/DSS; ^$$$$^*p* < 0.0001 *vs.* AOM/DSS + WT CD4^+^ T cells. The representative images of immunohistochemistry staining of Ki67 **(L)** in the colon of WT untreated mice, WT mice injected with AOM and treated with DSS and WT mice with induced colitis-associated colon cancer treated with WT or *Adgrf5* KO CD4^+^ T cells (*n* = 5 samples per group). The expression of *Il-1β***(M)** and *Tnf-α***(N)** at the mRNA level in in the colon of WT untreated mice, WT mice injected with AOM and treated with DSS and WT mice with induced colitis-associated colon cancer treated with WT or *Adgrf5* KO CD4^+^ T cells (*n* = 5 samples per group). Data are means ± SEM; ∗*p* < 0.05 *vs.* control; ^###^*p* < 0.001 *vs.* AOM/DSS; ^$$$^*p <* 0.001 *vs.* AOM/DSS + WT CD4^+^ T cells. The disease activity index score **(O)**, inflammation area **(P)** and spleen weight **(Q)** of untreated and DSS-treated WT and *Adgrf5* KO mice (*n* = 10 mice per group). The expression of *Il-2* (**R**), *IFN-γ***(S)**, *Tnf-α***(T)**, *Tbx21***(U)** and *Gata3***(V)** at the mRNA level in the colon of untreated and DSS-treated WT and *Adgrf5* KO mice (*n* = 5 mice per group). Data are presented as means ± SEM; ∗*p* < 0.05, ∗∗*p* < 0.01, ∗∗∗*p* < 0.001, ∗∗∗∗*p* < 0.0001 *vs.* control; ^#^*p* < 0.05, ^##^*p* < 0.01, ^###^*p* < 0.001 *vs.* DSS–WT mice.

To further examine the role of *Adgrf5* in colon tumors, we induced colitis-associated colon cancer (CAC) in WT and *Adgrf5* KO mice (Supplementary data). As shown in Figure 1H, *Adgrf5* KO mice injected with azoxymethane (AOM) and treated with dextran sulfate sodium (DSS) died before day 40 of the experiment, while only one death occurred among in WT mice with CAC. WT mice with CAC treated with *Adgrf5* KO CD4^+^ T cells were characterized by poor survival compared to WT mice without CAC and WT mice with CAC treated with WT CD4^+^ T cells (Fig. 1H). The non-treated animals with CAC and animals with CAC treated with WT CD4^+^ T cells had not yet developed tumors by day 63, while mice receiving *Adgrf5* KO CD4^+^ T cells developed multiple tumors (Fig. 1I). Colons obtained from mice with CAC treated with *Adgrf5* KO CD4^+^ T cells exhibited a significantly higher macroscopic damage score and inflammation length than colons from mice with CAC and mice with CAC treated with WT CD4^+^ T cells (Fig. 1J and K). A strong expression of the proliferation marker Ki67 in the colon crypts and surrounding structures in the colon of mice with CAC treated with *Adgrf5* KO CD4^+^ T cells was detected, confirming that *Adgrf5* KO CD4^+^ T cells cause colon tumor formation related to colitis (Fig. 1L). It is well known that T helper (T_h_) 1 cells may drive colitis, and a wide spectrum of pro-inflammatory cytokines, including IL-1β and tumor necrosis factor α (TNF-α), seem to be directly linked to the pathogenesis of colitis.^3^^,^^4^ To explore the impact of ADGRF5 on colitis development, we assessed the expression of the above-mentioned pro-inflammatory cytokines in the colons obtained from mice with CAC. Both investigated genes were overexpressed (Fig. 1M and N) in the colons of mice following *Adgrf5* KO CD4^+^ T cells treatment compared to animals with CAC and those treated with WT CD4^+^ T cells.

Since we found a dual function of ADGRF5 in colon cancer depending on the molecular context, we next examined the impact of ADGRF5 in the murine model of colitis induced by DSS administration. To specify the significance of ADGRF5 in the immune response, we administered DSS to WT and *Adgrf5* KO mice (Supplementary data). As expected, the disease activity index for *Adgrf5* KO mice with colitis was significantly enhanced compared to WT and *Adgrf5* KO mice without colitis and WT mice with colitis (Fig. 1O). Moreover, macroscopic evaluation of colons and spleens documented that *Adgrf5* KO mice with colitis had an increased area of inflammation and higher weight of spleen compared to WT mice with colitis (Fig. 1P, Q). Finally, we characterized the control and inflamed colons of WT and *Adgrf5* KO mice by real-time PCR and detected overexpression of *Il-2*, *Ifn-γ,* and *Tnf-α* (Fig. 1R–T) at the mRNA level in the inflamed colon of *Adgrf5* KO mice compared to the non-inflamed colons of *Adgrf5* KO mice and inflamed colons obtained from WT mice. Since CD4^+^ T cells with *Adgrf5* KO were characterized as a T_h_1-like population, we evaluated *Tbx21* (T-box transcription factor 21) and *Gata3* (GATA-binding protein 3) expression in the colon of mice with colitis, as these are the main markers of T_h_1 and T_h_2 cells, respectively. We found that *Tbx21* expression was increased while *Gata3* expression was decreased in the colons obtained from *Adgrf5* KO mice with colitis compared to the inflamed colons of WT mice (Fig. 1U and V). These findings collectively indicate that *Adgrf5* KO induces a T_h_1-like immune response in the colon and is responsible for colitis progression.

Taken together, our results indicate that CD4^+^ T cells with *Adgrf5* KO have the opposite effect in colitis and CAC compared to the MC38 cells-derived allograft tumor model. This dichotomy seems to be related to the secretion of pro-inflammatory cytokines, which prevent the proliferation of colon cancer cells but boost colitis development in chemically induced colon cancer and colitis model. Additionally, the genome of MC38 cells-derived allograft tumors but not tumors from CAC is described as microsatellite unstable and can be associated with the efficiency of adoptive transfer of CD4^+^ T cells, which is further elaborated in Supplementary data. Overall, our results suggest that ADGRF5 plays a critical role in the progression of colon cancer and colitis. ADGRF5 expression and its signaling pathways determine the function of CD4^+^ T cells, whose action is related to the T_h_1-like phenotype. In fact, previous data documented that T_h_1 cell infiltration corresponds to a better prognosis of CRC patients, while the T_h_1 secretome promotes the development of chronic inflammation.^5^ Nevertheless, it has to be mentioned that ADGRF5 is expressed not only in CD4^+^ T cells, but also in epithelial cells. However, we found that the protein level of ADGRF5 in the non-inflamed and inflamed colon of WT mice (Supplementary data) is comparable, suggesting its essential role in CD4^+^ T cells during the progression of colon cancer and colitis. Furthermore, these results demonstrate that ADGRF5 may be a target for novel therapeutics for colon cancer, CAC, and inflammatory bowel diseases. However, our findings suggest that CD4^+^ T cell-based immunotherapy targeting ADGRF5 may be effective for patients with microsatellite instability colon cancer. Conversely, modulating ADGRF5 activity in CD4^+^ T cells by specific agonists may yield therapeutic benefits in microsatellite stable colon cancers and colitis. Nonetheless, further studies providing direct evidence to validate these findings are warranted.

## CRediT authorship contribution statement

**Damian Jacenik:** Writing – review & editing, Writing – original draft, Resources, Project administration, Methodology, Investigation, Funding acquisition, Formal analysis, Data curation, Conceptualization. **Marta Zielińska:** Investigation. **Federica Ungaro:** Writing – review & editing. **Silvio Danese:** Writing – review & editing. **Ellen J. Beswick:** Investigation, Formal analysis. **Jakub Fichna:** Writing – review & editing, Writing – original draft.

## Ethics declaration

The experimental protocol was approved by the Local Ethical Committee at the Medical University of Lodz (ŁB236-UZ-B/2024, 23/ŁB236/2022, 5/ŁB156/2020 and 43/ŁB153/2019).

## Data availability

The datasets generated and/or analyzed during the current study are available from the corresponding author on reasonable request.

## Funding

This work was supported by a grant (2019/32/C/NZ5/00064 to DJ) from the National Science Centre, Poland.

## Conflict of interests

The authors declare no competing interests.
