# Supplementary material for: The adhesion G protein-coupled receptor F5 is a crucial regulator of CD4+ T cell function: Implications for colon cancers and colitis progression
Source: Genes Dis. 2026 Feb 23;14(2):102107. doi: 10.1016/j.gendis.2026.102107 (PMC13654459; doi:10.1016/j.gendis.2026.102107)
Supplement: Supplementary file 1 [file mmc1.docx]

**SUPPLEMENTARY DATA**

**Results**

**Supplementary Figure 1. Representative flow cytometry gating plots using for purity determination of CD4^+^ T cells isolated from murine spleens.** On the left side unstained (blue) and stained splenocytes using PE-CD4^+^ antibodies (red). On the right side unstained purified CD4^+^ T cells (blue) and purified CD4^+^ T cells stained with PE-CD4^+^ antibodies (red).

**Supplementary Figure 2.** Representative image from Western blot analysis showing the level of ADGRF5 in the proximal non-inflamed (control) and inflamed (2.5% DSS) colon obtained from WT mice. The lack of ADGRF5 expression differences between uninflamed and inflamed colon, our data suggests that CD4^+^ T cells play a pivotal role in the modulation of colitis.

**Materials and methods**

***Animals***

C57BL/6 and *Adgrf5* KO mice were obtained from Jackson Laboratory (Bar Harbor, MA, USA) and Dr. Brad St. Croix (National Cancer Institute, Frederick, MD, USA) and were bred in-house. The animals were housed in the Animal Facility of the University of Lodz, Poland at constant temperature (22 – 24°C), relative humidity ~55% and maintained under 12 h light/dark cycle with access to standard chow pellets and tap water *ad libitum*. The experimental protocol was approved by the Local Ethical Committee at the Medical University of Lodz (ŁB236-UZ-B/2024, 23/ŁB236/2022, 5/ŁB156/2020 and 43/ŁB153/2019).

***Isolation of CD4^+^ and CD8^+^ T cells***

T cells were isolated from spleens of 6 – 8 week-old WT and *Adgrf5* KO mice using EasySep^TM^ Mouse CD4^+^ T cell Isolation Kit and EasySep^TM^ Mouse CD8^+^ T cell Isolation Kit. CD4^+^ and CD8^+^ T cells for *in vivo* experiments were directly injected to mice while for *in vitro* experiments were incubated in RPMI 1640 medium supplemented with 10% heat-inactivated fetal bovine serum, 1% penicillin/streptomycin and 1% L-glutamine. Some CD4^+^ and CD8^+^ T cells were activated with Dynabeads™ Mouse T Activator CD3/CD28, incubated for up to 24 h, and collected for multiplex and real-time PCR analysis. Flow cytometry was used to confirm the phenotype of CD4^+^ and CD8^+^ T cells directly after isolation. T cells were collected and washed using phosphate-buffered saline (PBS) containing 1% bovine serum albumin. The supernatant was removed and T cells were incubated with 2% rat serum and antibody against CD4 (clone GK1.5) or CD8 (clone 53-6.7). Data was collected using BD FACSymphony^TM^ A1Cell Analyzer and analyzed with BD FACSymphony^TM^ A1Cell Analyzer Software.

***Murine allograft of colon tumor model and treatments***

MC38 cells were cultured in complete RPMI 1640 medium and used to generate a murine allograft model of colon tumor model. 5 × 10^5^ of MC38 cells resuspended in PBS, mixed (v/v) with Matrigel^®^ and were injected into the right flank of 6 – 8 week-old male WT and *Adgrf5* KO mice. Some WT mice with MC38 cells-derived allograft colon tumor were administrated peritumorally (*i.t.*) with 1 × 10^6^ of WT or *Adgrf5* KO CD4^+^ T cells or 100 µg of antibody against IFN-γ or isotype control in total volume 100 uL. Mice without treatment received 100 uL of PBS. Tumors were manually measured using caliper starting from day 1. Tumor size was calculated according to the following formula: tumor size = (length × length × width)/2.

***Murine model of colitis-associated colon cancer and treatments***

Colitis-associated colon cancer was induced in WT and *Adgrf5* KO mice by a single intraperitoneal (*i.p.*) injection of azoxymethane (AOM) at a dose of 7.5 mg/kg. One week later autoclaved water was replaced by 1% (w/v) dextran sodium sulfate (DSS) solution. Further, after one week DSS was replaced by autoclaved water for the next two weeks and this cycle was then repeated two more times. In addition, some WT mice with colitis-associated colon cancer were administrated with 5 × 10^5^ of WT or *Adgrf5* KO CD4^+^ T cells once per week. To note, animals without treatment received 100 uL of PBS. Body weight, health, and general behavior of animals were monitored once a day. Mouse spleens and colons were separated, washed, and weighted and the macroscopic damage score was performed.

***The macroscopic score – murine model of colitis-associated colon cancer***

The total macroscopic damage score was calculated for each animal based on the following parameters: presence of diarrhea (from 0 to 3 points), the extent of colon damage (from 0 to 3 points), colon length score (0 points for shortening less than 5%, 1 point for shortening between 5 and 14%, 2 points for shortening between 15 and 24% shortening, 3 points for shortening between 25 and 35%, 4 points for shortening higher than 35% as compared to control group) and colon weight score (0 points for weight loss than 5%, 1 points for weight loss between 5 and 14%, 2 points for weight loss between 15 and 24%, 3 points for weight loss between 25 and 35%, 4 points for weight loss higher than 35% as compared to control group). The presence (1 point) or absence (0 points) of fecal blood was also included into the macroscopic damage score.

***Murine model of colitis***

Colitis was induced by DSS administration into drinking autoclaved water (2.5% w/v) for 5 consecutive days. After that, DSS was replaced by autoclaved water. Body weight, stool consistency, and blood in feces of animals were monitored and disease activity index based on weight score (0 points for lack of weight change, 1 points for weight loss between 1 and 5%, 2 points for weight loss between 6 and 10%, 3 points for weight loss between 11 and 20%, 4 points for weight loss higher than 20% as compared to control group), stool consistency score (0 points for regular consistency, 2 points for loose stool, 4 points for diarrhea) and presence of blood in faces (0 points for lack of blood, 2 points for occult bleeding, 4 points for gross bleeding) was evaluated. Animals were sacrificed at day eight and both colon and spleen were separated, washed, weighted and the macroscopic damage score was performed.

***The macroscopic score – murine model of colitis***

The macroscopic damage score is a sum of the following parameters: colon length score (0 points for shortening less than 5%, 1 point for shortening between 5 and 14%, 2 points for shortening between 15 and 24% shortening, 3 points for shortening between 25 and 35%, 4 points for shortening higher than 35% as compared to control group), colon weight score (0 points for weight loss than 5%, 1 points for weight loss between 5 and 14%, 2 points for weight loss between 15 and 24%, 3 points for weight loss between 25 and 35%, 4 points for weight loss higher than 35% as compared to control group), the presence (1 point) or absence (0 points) of erythema and fecal blood, intensity of diarrhea (from 0 to 3 points) and bowel thickness.

***Histology analysis***

Colon samples were dehydrated, embedded in paraffin, sectioned at 5 μm using Leica RM2255 fully automated rotary microtome, and mounted onto slides. The sections were stained with hematoxylin and eosin and examined. Photographs were taken using hardware (AX-70 light microscope, Imaging Solutions Camera) and software (Cell F Imaging Software) from Olympus and Olympus Soft Imaging Solutions, respectively.

***Western blot analysis***

Proteins were isolated in the radioimmunoprecipitation assay buffer using an ultrasonic homogenizer. The homogenates were cleared by centrifugation at 5,000 rpm for 10 minutes. Total protein concentration was evaluated in each sample in triplicate using the Lowry protocol. 30 μg of protein samples were separated on 12% polyacrylamide gels in electrophoresis buffer (25 mM Tris, pH = 8.3; 192 mM glycine; 0.1% SDS) and electrotransferred in transfer buffer (25 mM Tris, pH = 8.3; 192 glycine; 20% methanol) using wet system onto polyvinyl difluoride membranes (pore size 0.45 µm). Membranes were blocked with 5% casein before incubation with commercially available primary antibodies against CD3E (cat. no. A199017) overnight at 4 °C. For each sample, a separate analysis was performed using β-actin antibodies (cat. no. #4970). Subsequently, membranes were incubated with secondary antibodies coupled with HRP. Immunoreaction was visualized using Clarity^TM^ Western ELC Substrate and X-ray films.

***Tumor-killing assay***

5 × 10^4^ of MC38 cells were plated, WT or *Adgrf5* KO CD4^+^ T cells in a ratio 1:2 were added and incubated for up to 24 h. Some CD4^+^ T cells in co-cultures were activated for up to 18 h with 1.25 µL of Dynabeads™ Mouse T Activator CD3/CD28 to obtain a bead-to-CD4^+^ T cell ratio of 1:1. Supernatants were collected for multiplex analysis while co-cultures were washed with PBS and flow cytometry analysis was performed using monoclonal antibody against CD4 (cat. no. #12-0041-82) and Apoptosis and Necrosis Assay Kit from MedChemExpress according to the manufacturer protocol. Samples were stained in duplicates and data collected using an Attune™ NxT Flow Cytometer and analyzed with Attune™ NxT Software.

***Immunohistochemistry analysis***

The formalin-fixed paraffin-embedded sections (5 µm) were deparaffinized, dehydrated, and incubated in boiling sodium citrate buffer to antigen unmasking. After incubation in block solution containing 5% goat normal serum, 50 mM Tris/HCl, pH = 7.5, and 150 mM NaCl the sections were incubated overnight at 4 °C with commercially available primary antibody against Ki67. Next, the sections were washed and incubated with Alexa Fluor^®^ 488 goat anti-rabbit secondary antibodies. The sections was mounted with ProLong^TM^ Diamond Antifade Mountant with DAPI. Leica TCS SP8 confocal microscope was used for imaging. Confocal microscopy analysis was performed in the Laboratory of Microscopic Imaging and Specialized Biological Techniques at the Faculty of Biology and Environmental Protection, University of Lodz.

***RNA extraction, reverse transcription and real-time PCR analysis***

Total RNA isolation was performed using commercially available TRI Reagent^®^. cDNA synthesis was performed with a High-Capacity cDNA Reverse Transcription Kit in accordance with the manufacturer’s protocol. Quantification of mRNA was performed using the real-time PCR method with specific primers. The reaction mixture consisted of cDNA, PowerUp^TM^ SYBR^TM^ Green Master Mix, forward and reverse primers (**Table S1**) as well as RNase-free water in total volume of 10 μl.

Cycle parameters were as follows: UDG activation at 50 °C for two minutes and Dual-Lock^TM^ DNA polymerase at 95 °C for two minutes, followed by 40 cycles of sequential incubations at 95 °C for 15 s and at 60 °C for 1 minute. Obtained results were normalized to the expression of *Hprt1* (hypoxanthine phosphoribosyltransferase) gene. All experiments were performed as triplicates. The real-time PCR was performed using in Master Realplex4s. The relative expression level was calculated as 2^−∆Ct^ × 1000. The results expressed as number of examined mRNA copies per 1000 copies of mRNA for *Hprt1*.

**Table S1. The sequence of primers used in real-time PCR analysis.**

| Gene abbreviation | Forward primer (5’ – 3’) | Reverse primer (5’ – 3’) |
| --- | --- | --- |
| *Gata3* | TATTAACAGACCCCTGACTATG | CACCTTTTTGCACTTTTTCG |
| *Gzmb* | GGACAAAGGCAGGGGAG | AAGTAAGGCCATGTAGGGTC |
| *Ifn-γ* | CTAGCTCTGAGACAATGAAC | CTCTTTTCTTCCACATCTATGC |
| *Il-1β* | GGATGATGATGATAACCTGC | CATGGAGAATATCACTTGTTGG |
| *Il-2* | AGGATGGAGAATTACAGGAAC | AGAGGTCCAAGTTCATCTTC |
| *Prf1* | TTGGTGGGACTTCAGCTTTC | GAAAAGGCCCAGGAGGAAC |
| *Tbx21* | ACGTCTTTACTTTCCAAGAG | GTACATGGACTCAAAGTTCTC |
| *Tnf-α* | CTATGTCTCAGCCTCTTCTC | CATTTGGGAACTTCTCATCC |
| *Hprt1* | AGGGATTTGAATCACGTTTG | TTTACTGGCAACATCAACAG |

***Multiplex analysis***

MC38 cells-derived allograft tumors were divided into 8 mg pieces (± 0.5 mg) and incubated in complete RPMI media up to 18 h. The supernatants from *in vitro* experiments and MC38 cells-derived allograft tumors were collected and analyzed for cytokine levels by multiplex arrays and Luminex^®^ in accordance with the manufacturer’s instructions.

***Statistical analysis***

Statistical analysis was performed using GraphPad Prism 5.0. Results are presented as means ± standard error of the mean (SEM). Parametric unpaired t-test, non-parametric Mann–Whitney *U*-test, one-way ANOVA followed by Tukey multiple comparison post hoc test and two-way ANOVA followed by Bonferroni’s multiple comparison post hoc test were used for comparison of studied groups. *p*-values < 0.05 was considered statistically significant.

**Discussion**

According to our findings ADGRF5 reprograms CD4^+^ T cells, and its deletion switches CD4^+^ T cells into a T_h_1-like phenotype. We show that these genetically modified CD4^+^ T cells are beneficial for the MC38 cells-derived allograft tumor model. Interestingly, we noted an opposite effect in the murine model of CAC. This phenomenon may be associated with the heterogeneity of colon cancer cells derived from the allograft colon tumor model and the chemically induced model of CAC and their tumor microenvironment (TME). The immune cell component of the tumor – including macrophages, mast cells, natural killer (NK) cells, T and B cells, among others – is fundamental in determining its ability to proliferate, invade and metastasize. Recent studies indicate that TME regulates not only neoplastic transformation or progression, but may affect the response to treatment in cancer patients^1^. Based on this evidence, numerous multi-omics approaches were developed to track TME and predict cancer progression and treatment efficiency. For instance, the immuno-oncology biological research (IOBR) package, the tumor immune microenvironment explorer (HPVTIMER) or tumor immune estimation resource (TIMER and TIMER 2.0) combines large datasets regarding gene expression signatures related to immune cells, gene mutation, pathways and correlation analysis as well as clinical data, among others^2-5^. More and more evidence highlights that T cells play an essential role in cancer progression, and dependent on their status they can eliminate target cancer cells or evoke signals promoting cancer cell proliferation. CD4^+^ T cells can differentiate into a number of subpopulations such as T_h_1, T_h_2, T_h_9, T_h_17, and T regulatory (T_reg_) cells. The above-mentioned subpopulations of T cells are classified based on the specific pattern of cytokines released by CD4^+^ T cells. For instance, T_h_1 cells are characterized by the secretion of IL-2, IFN-µ and TNF-α, which are responsible for cancer cell death promotion, inhibition of angiogenesis, and boosting other immune cells such as macrophages and natural killer (NK) cells. T_h_1 cells may affect apoptosis of cancer cell directly; however indirect action involving cytokines related to T_h_1 cells which enhance immune response has been documented. On the other hand, T_h_2 or T_reg_ cells release anti-inflammatory cytokines such as IL-10 or transforming growth factor β, which suppress anti-cancer response of T_h_1 cells and promote immunosuppressive microenvironmental leading to cancer promotion. The infiltration signature of CD4^+^ T cell subpopulations in colon cancer seems to be useful in the prediction of patients outcomes. Additionally, the estimation of immune cell infiltration in colon cancer using multi-omics approaches may be beneficial in the tracking of treatment efficiency.

Our study documented that *Adgrf5* KO CD4^+^ T cells secrete cytokines associated with T_h_1 cells, which inhibit the growth of MC38 cells-derived allograft tumors when compared to the WT CD4^+^ T cells. In contrast, genetically modified CD4^+^ T cells promote colitis which seems to be directly related to opposite effect of cytokines such as IFN-µ and TNF-α in colon cancer and colitis settings. However, our further findings documented that *Adgrf5* KO CD4^+^ T cells promote colon cancer progression in CAC model. This discrepancy between MC38 cells-derived allograft tumor model and CAC model may be associated with molecular differences between these two models which include for instance post-transcriptional regulation, methylation status or gene variations. In fact, MC38 cells are widely used in preclinical studies to induce of murine model of colon cancer, especially for immunotherapies, which seems to be related to the presence of several immunogenic neo-epitopes responsible for interaction with cells^6^. Additionally, whole-genome and RNA sequencing identified 5931 somatic mutations in MC38 cells including specific mutations in DNA mismatch-repair (MMR) genes, which mimic microsatellite instability (MSI) in human colon cancers^7^. In contrast, in the colon obtained from the murine model of CAC, 2986 somatic mutations affecting protein coding sequence were found. Interestingly, low frequencies of mutations in MMR genes were noted, which suggests that CAC can be described as model of microsatellite stable (MSS) colon cancer^8^. It was documented that both subtypes of colon cancer, i.e. MSI and MSS have a different response rate to immunotherapy. In fact, MSI colon cancers are much more prone to immunotherapy, and this phenomenon is associated with high accumulation rate of mutations leading to formation of frameshift proteins and neo-antigens, which are recognized by immune cells. These findings seem to be directly related to our observation that *Adgrf5* KO CD4+ T cell treatment abolished colon cancer progression in MC38 cells-derived allograft tumor model but not in chemically induced model of CAC.

Taken together, our experimental data highlight that ADGRF5 is an essential receptor for CD4^+^ T cell function, and modulation of its expression determines the phenotype of this population of immune cells. Additionally, we found a bidirectional function of *Adgrf5* KO CD4^+^ T cells: it abolishes progression of colon cancer characterized by MSI but is not an effective treatment approach for colon cancers with MSS such as CAC. In fact, secretome of *Adgrf5* KO CD4^+^ T cells, which is beneficial for colon cancer, seems to be responsible for the development of colitis. Our data suggests that ADGRF5-targeted immunotherapy employing agonists and antagonist may serve a new treatment strategy for colon cancer patients and patients with inflammatory bowel diseases such as Crohn’s disease and ulcerative colitis. Nevertheless, additional effort combining drug discoveries and multi-omics approaches has to be made to explore potent and specific ligands of ADGRF5.

**References**

1. Almazrouei KM, Mishra V, Pandya H, Sambhav K, Bhavsar SN. Tumor Microenvironment and Its Role in Cancer Progression: An Integrative Review. *Cureus*. Sep 2025;17(9):e92707. doi:10.7759/cureus.92707

2. Zeng D, Ye Z, Shen R, et al. IOBR: Multi-Omics Immuno-Oncology Biological Research to Decode Tumor Microenvironment and Signatures. *Front Immunol*. 2021;12:687975. doi:10.3389/fimmu.2021.687975

3. Liu L, Xie Y, Yang H, et al. HPVTIMER: A shiny web application for tumor immune estimation in human papillomavirus-associated cancers. *Imeta*. Aug 2023;2(3):e130. doi:10.1002/imt2.130

4. Li T, Fan J, Wang B, et al. TIMER: A Web Server for Comprehensive Analysis of Tumor-Infiltrating Immune Cells. *Cancer Res*. Nov 1 2017;77(21):e108-e110. doi:10.1158/0008-5472.can-17-0307

5. Li T, Fu J, Zeng Z, et al. TIMER2.0 for analysis of tumor-infiltrating immune cells. *Nucleic Acids Res*. Jul 2 2020;48(W1):W509-w514. doi:10.1093/nar/gkaa407

6. Yadav M, Jhunjhunwala S, Phung QT, et al. Predicting immunogenic tumour mutations by combining mass spectrometry and exome sequencing. *Nature*. Nov 27 2014;515(7528):572-6. doi:10.1038/nature14001

7. Efremova M, Rieder D, Klepsch V, et al. Targeting immune checkpoints potentiates immunoediting and changes the dynamics of tumor evolution. *Nat Commun*. Jan 2 2018;9(1):32. doi:10.1038/s41467-017-02424-0

8. Pan Q, Lou X, Zhang J, et al. Genomic variants in mouse model induced by azoxymethane and dextran sodium sulfate improperly mimic human colorectal cancer. *Sci Rep*. Dec 2017;7(1):25. doi:10.1038/s41598-017-00057-3
